# Cyclic adenosine monophosphate-regulated transcriptional co-activator 3 polymorphism in Chinese patients with acute coronary syndrome

**DOI:** 10.1097/MD.0000000000011382

**Published:** 2018-07-06

**Authors:** Li Zhu, Yahui Wang, Jun Jiang, Ruifang Zhou, Jun Ye

**Affiliations:** a Department of Laboratory Medicine, Taizhou People's Hospital; b Taizhou Polytechnic College, Taizhou, China.

**Keywords:** acute coronary syndrome, cyclic adenosine monophosphate-regulated transcriptional co-activator 3, cyclic adenosine monophosphate-responsive element-binding protein, polymorphism

## Abstract

To investigate the cAMP-regulated transcriptional co-activator 3 **(**CRTC3) polymorphism and its significance in the acute coronary syndrome patients.

In total, 248 patients with acute coronary syndrome admitted to Taizhou People's Hospital between March 2016 and October 2016 were included in this study. Eighty-eight age- and gender-matched healthy individuals received physical examination in our hospital served as normal control. Single nucleotide polymorphism (SNP) analysis of CRTC3 (rs3862434 and rs11635252) was evaluated using PCR amplification.

For the SNP of CRTC3, significant differences were identified in rs3862434 (AA/AG) and rs11635252 (TT/CT/CC) between the 2 groups (*P* < .05). Statistical increase was noticed in the high density lipoprotein cholesterol (HDL-C) in those with AG phenotype compared with those with AA phenotype in those with rs3862434. Significant decrease was identified in the total cholesterol (TC), triglyceride (TG), and weight in those with CC phenotype compared with those with CT phenotype among the cases with rs11635252 (*P* < .05).

CRTC3 polymorphism was associated with the onset of acute coronary syndrome in Han Chinese patients, which may be related to the imbalance of the lipid metabolism.

## 1 Introduction

Coronary atherosclerotic heart disease, also named coronary heart disease (CHD), is the major cause for cardiovascular related mortality in developed countries.^[1]^ These patients usually present angina pectoris, myocardial infarction, and even sudden death due to lumen stenosis induced blood insufficiency in myocardium.^[2]^ To date, the pathogenesis of CHD has been related to be several conditions including obesity, hyperlipidemia, and diabetes mellitus.

Cyclic adenosine monophosphate (cAMP)-responsive element-binding protein (CREB) is phosphorylated in response to several signals, while in some cases, target gene transcription is only increased. Recently, several studies indicate that CREB functions in concert with a family of latent cytoplasmic co-activators called cAMP-regulated transcriptional co-activators (CRTCs), which are activated via dephosphorylation. *CRTC3* expression is reported to be associated with the metabolism of saccharide and lipid.^[3,4]^ Meanwhile, soluble *CRTC3* polymorphism is related to the pathogenesis of obesity.^[5,6]^ In this study, we aim to investigate the single nucleotide polymorphism (SNP) of *CRTC3*, *rs3862434,* and *rs11635252* in patients with acute coronary syndrome, and explore its correlation with the urea nitrogen, creatinine, uric acid, total cholesterol (TC), triglyceride (TG), blood glucose, low density lipoprotein cholesterol (LDL-C), and high density lipoprotein cholesterol (HDL-C).

## 2 Materials and methods

### 2.1 Subject

A total of 248 patients (male: 181, female: 67; mean age, 65.71 ± 11.51 years) with acute coronary syndrome admitted to Taizhou People's Hospital between March 2016 and October 2016 were included in this study. Eighty-eight age- and gender-matched healthy individuals (male: 58, female: 30, mean age, 63.11 ± 7.26 years) received physical examination in our hospital served as normal control. Those with severe liver and renal insufficiency, infectious diseases, hematological diseases, rheumatic immune disease, and malignancy were excluded from this study. Each patient signed the informed consent. The study protocols were approved by the Ethical Committee of Taizhou People's Hospital.

### 2.2 Primer design

*CRTC3* gene sequence (NG_ 012808.1) was downloaded from the GeneBank database (http://www.ncbi.nlm.nih.gov/nucleotide/257196128?report=genbank&log$=nucltop&blast_rank=1&RID=X5AA8UC801S). The primers listed in Table 1 were designed using Primer 5.0 software; PREMIER Biosoft International, Palo Alto CA based on the downloaded sequence. Two sites (i.e. *rs3862434* and *rs11635252*) were utilized in the SNP analysis.

**Table 1:**
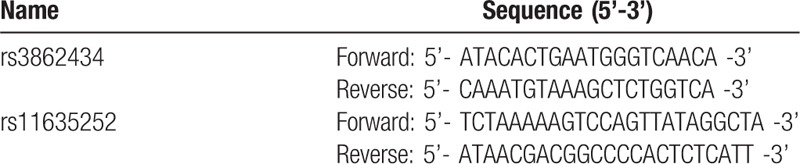
Sequences of primer.

### 2.3 Blood sample extraction

Venous blood was collected from each patient in a fasting state using Eppendorf tube containing edathamil (EDTA-K2). Sample collection was carried out using Blood Gen Mini Kit (CwBiotech, Beijing, China), according to the manufacturer's instructions.

### 2.4 SNP of *CRTC3* gene

SNP analysis of *rs3862434* and *rs11635252* was evaluated using PCR amplification. PCR amplification was carried out in a total volume of 50 μL containing 10 × Tag Buffer, 2.5 mM dNTP, 50pM each primer, 1 μl DNA Taq polymerase (Futai Biotech, Nanjing, China) and 5 μL DNA template. The amplification conditions were as follows: pre-denaturation at 95°C for 5 minutes, followed by 35 cycles of 94°C for 30 seconds, 55°C for 30 seconds, and 72°C for 30 seconds. The obtained PCR products were subject to Sanger Sequencing.

Determination of blood urea nitrogen (BUN), creatinine (CRE), uric acid (UA), TC, triglyceride, blood glucose (GLU), LDL-C, and HDL-C.

Venous blood was collected from the patients about 24 hours after admission to detect the BUN, CRE, UA, TC, TG, GLU, LDL-C, and HDL-C. BUN, CRE, UA, TC, TG, and GLU were determined using commercial enzyme linked immunosorbent assay (ELISA) kits, according to the manufacturer's instructions. LDL-C and HDL-C were determined using automatic analyzer. All the tests were performed at least in triplicate.

### 2.5 Statistical analysis

Data analysis was carried out using SigmaPlot 12.0 software; Systat Software Inc. (SSI), San Jose, California. Data normally distributed were presented as mean ± standard deviation. Student *t* test was used for the inter-group comparison. The data of nonnormal distribution was presented as (median, 25%–75%), and were analyzed using the rank test. *P* < .05 was considered to be statistically significant.

## 3 Results

### 3.1 SNP comparison

**Figure 1:**
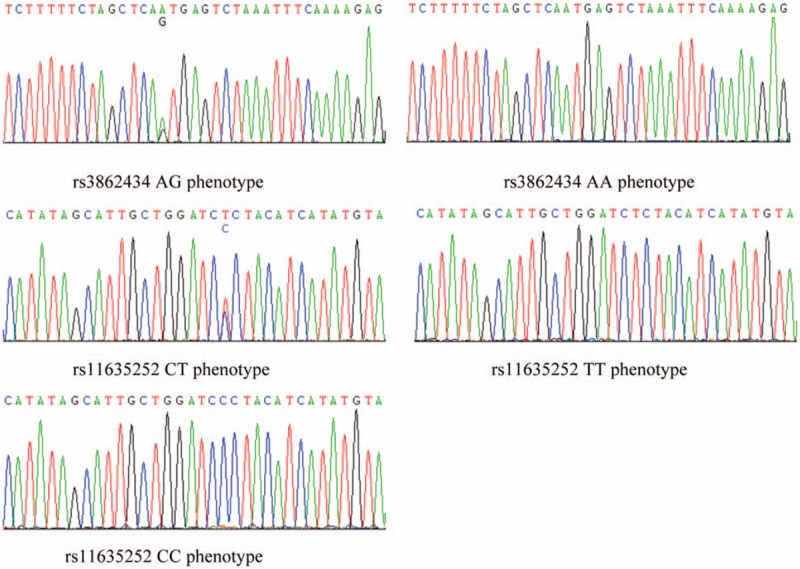
SNP of rs3862434 and rs11635252 of CRTC3 gene.

No statistical differences were noticed in the sex, age, and LDL-C between the acute coronary syndrome group and the normal control (*P* > .05). In contrast, statistical differences were observed in the BUN, CRE, UA, GLU, TC, TG, and HDL-C between the 2 groups (*P* < .05, Table 2). For the SNP of CRTC3, significant differences were identified in rs3862434 (AA/AG) and rs11635252 (TT/CT /CC) between the 2 groups (*P* < .05, Fig. 1).

**Table 2:**
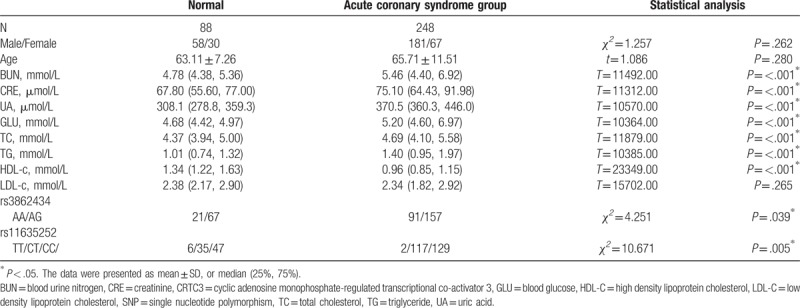
Comparison of clinical data and CRTC3 SNP.

Comparison of BUN, CRE, UA, GLU, TC, TG, HDL-C, LDL-C, weight, and BMI in *rs3862434* and *rs11635252* of *CRTC3* in patients with acute coronary syndrome

No differences were noticed in the BUN, CRE, UA, GLU, TC, TG, HDL-C, LDL-C, weight, body mass index (BMI), and CRTC3 SNP in different phenotypes in *rs3862434* and *rs11635252* of *CRTC3* of normal group (Table 3). Our data showed that the BMI and weight showed no statistical differences were noticed in the AG phenotype and AA phenotype of rs3862434. For the CC phenotype and CT phenotype of rs11635252, no statistical differences were noticed in the BMI, but statistical differences were noticed in the body weight. No statistical differences were observed in BUN, CRE, UA, GLU, LDL-C, and BMI between the AA and AG phenotype in those with *rs3862434*, as well as between CT and CC phenotype in those with *rs11635252*, respectively (*P* > .05, Table 4). Nevertheless, statistical increase was noticed in the HDL-C in those with AG phenotype compared with those with AA phenotype in those with *rs3862434*. Significant increase was identified in the TC, TG, and weight in those with CT phenotype compared with those with CC phenotype among the cases with *rs11635252* (*P* < .05).

**Table 3:**
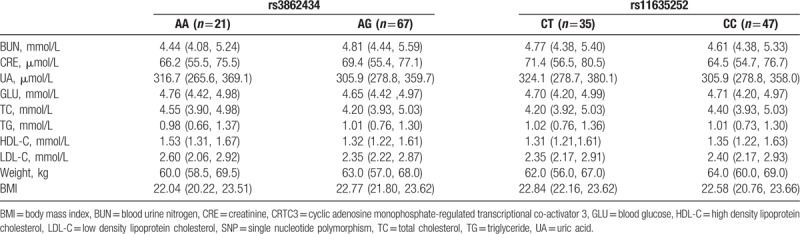
Comparison of BUN, CRE, UA, GLU, TC, TG, HDL-C, LDL-C, weight, BMI, and *CRTC3* SNP in different phenotypes in normal group.

**Table 4:**
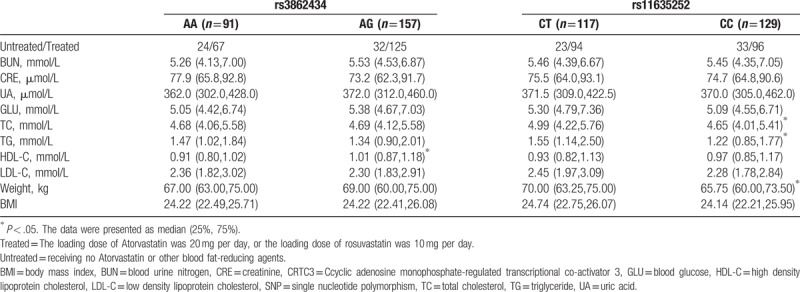
Comparison of BUN, CRE, UA, GLU, TC, TG, HDL-C, LDL-C, weight, BMI, and *CRTC3* SNP in different phenotypes in acute coronary syndrome group.

## 4 Discussion

The onset of coronary heart disease is reported to be associated with several factors such as imbalance of lipid metabolism, insulin resistance and obesity. In the present study, we investigate the SNP of *CRTC3*, *rs3862434,* and *rs11635252* in patients with acute coronary syndrome (ACS). Also, we explored its correlation with the urea nitrogen, creatinine, uric acid, TC, TG, blood glucose, HDL-C, and LDL-C.

CREB family and coactivator CRTC protein played important roles in the insulin sensitivity.^[7]^ In a previous study, CREB and CRTC protein family contributed to the expression of downstream genes of c-AMP response element (CRE) such as PPARγ, PGC-lα mRNA, as well as the transcription and expression of downstream genes of mitochondrial respiratory chain and TCA cycle.^[8]^ On this basis, it may contribute to the metabolism of lipids and energy, which then regulates the balance between lipids and energy.

As an important member of CREB coativator, CRTC3 was highly expressed in the white blood cells and fats, which could affect the lipid metabolism and contribute to the development of obesity through modulating the catecholamine signal in the adipose tissues.^[9]^ According to the previous studies, CRTC3 was closely involved in the lipolysis, biosynthesis in mitochondria, fatty acid oxidation, as well as lipid metabolism.^[10–12]^ Compared with the wild type rats, CRTC3 gene knock-out triggered elevation of energy consumption and increase of oxidant, which showed significant responses in the insulin sensitivity test, together with reduction of adipose tissues and body weight. Besides, *rs8033595* and *rs3862434* were associated with the risk of overweight in Mexican-Americans, rather than non-Hispanic whites.^[4]^ Bachman et al^[9]^ reported that *rs8033595* of *CRTC3* was correlated with body weight, BMI, and hip circumference in the Mexican-Americans. Meanwhile, it could be correlated with the point mutation and the risk of overweight, especially in those with homozygous genotypes compared to those with heterozygous mutations. For the mechanism, it might be related to the fact that CRTC3 mutations may contribute to the transcription activity of G-protein regulatory factor 2 (RGS2) compared to the wild type. This indicated that *CRTC3* polymorphism might affect the energy metabolism through modulating the transcriptional activity of RGS2. Another SNP *rs3862434* showed high linkage disequilibrium with *rs8033595*, and similar association was noticed in the risk of obesity and Mexican-Americans. However, rs3862434 polymorphism showed no correlation with the risk of obesity in the non-Hispanic whites, which might be related to the environmental factors, life style and gene background. CRTC3 could affect the fat metabolism and contribute to the obesity in the adipose tissues through modulating the catecholamine.^[9]^ Our study indicated that the proportion of AA phenotype of CRTC3 *rs3862434* in patients with acute coronary syndrome was significantly higher than that of the normal control. Statistical increase was noticed in the HDL-c in those with AG phenotype compared with those with AA phenotype in those with *rs3862434*. Significant increase was identified in the TC, TG and Weight in those with CT phenotype compared with those with CC phenotype among the cases with rs11635252 (*P* < .05). All these implied that *rs3862434* AA phenotype and *rs11635252* CT phenotype were the risk factors for the pathogenesis of acute coronary syndrome. For the mechanism, these 2 sites could affect the balance of the lipid metabolism, which then triggered the onset of acute coronary syndrome. LDL-C played important roles in the pathogenesis and progression of CHD. In this study, there were no statistical differences in the LDL-C in patients and normal control. Meanwhile, there were no statistical differences between LDL-C in those of various genotypes. This may be related to the fact that part of the patients received administration of statins for the treatment.

The cAMP-PKA signaling pathway is closely involved in modulating the activity of CREB and the CRTCs. It could regulate the CREB and CRTCs through the phosphorylation and dephosphorylation processes. Besides, it mediated the transcription of downstream genes through inducing CRTCs. The cAMP or Ca^2+^ triggered the dephosphorylation of CRTC, as well as the nuclear translocation of CRTC, which contributed to the binding of CREB and the promoter. In adipose tissues, liver and skeletal muscles, CREB and the CRTCs could modulate the metabolism of sugar and lipids.^[13]^ With the synergistic activation function, CRTCs could stimulate the expression of *CRE* genes.^[14,15]^ CRTCs could bind with the ZIP DNA domain of the CREB, which significantly promoted the transcription of CRE related genes, such as BDNF and PGC-1α.^[16]^ In addition, CRTC promoted the binding between TAFII130 domain and CREB, which then led to increased transcription of Q2 region abundant with glutaminate.^[17,18]^ Moreover, the binding of CRTCs and CREB contributed to the mRNA expression of PPARγ and PGC-1α, which was crucial for the metabolism of lipids and energy.

There are really limitations in our study. The study is a cross-sectional study. Besides, the sample size was not large enough. Moreover, there is a lack of ethnic diversity in this study, and potential errors may present in the sequencing process although this is likely a much lesser concern. In future, large sample size studies are needed to validate our results. Despite the fact that we proved CRTC3 polymorphism was associated with the onset of acute coronary syndrome in Han Chinese patients, we cannot find the exact mechanism for it up to now. Such process may be related to the imbalance of the lipid metabolism. In future, we will focus on the in vivo and in vitro experiments to further identify the mechanisms.

In conclusion, *CRTC3* polymorphism was associated with the onset of acute coronary syndrome in Han Chinese. In future, further studies are needed to illustrate the exact mechanism.

## Author contributions

**Conceptualization:** Jun Ye.

**Data curation:** Ruifang Zhou, Jun Ye.

**Formal analysis:** Ruifang Zhou.

**Methodology:** Yahui Wang.

**Software:** Jun Jiang, Ruifang Zhou.

**Validation:** Jun Jiang, Ruifang Zhou.

**Visualization:** Jun Jiang, Ruifang Zhou.

**Writing – original draft:** Li Zhu.

**Writing – review & editing:** Jun Ye.

## References

[1] Engelbertz C Reinecke H Breithardt G Two-year outcome and risk factors for mortality in patients with coronary artery disease and renal failure: the prospective, observational CAD-REF Registry. Int J Cardiol 2017;243:65–72. 28526542 10.1016/j.ijcard.2017.05.022

[2] Wu Y Zhang XX Tian L Impact of CYP2C19 genotype and platelet function on clinical outcome in coronary atherosclerotic heart diseases patients received clopidogrel post percutaneous coronary intervention. Zhonghua Xin Xue Guan Bing Za Zhi 2017;45:377–85. 28511321 10.3760/cma.j.issn.0253-3758.2017.05.004

[3] Micic D Polovina S Obesity and coronary heart disease: the mechanism of atherogenic impact. Med Pregl 2009;62:43–6. 19702115

[4] Song Y Altarejos J Goodarzi MO CRTC3 links catecholamine signalling to energy balance. Nature 2010;468:933–9. 21164481 10.1038/nature09564 PMC3025711

[5] Prats-Puig A Soriano-Rodriguez P Oliveras G Soluble CRTC3: a newly identified protein released by adipose tissue that is associated with childhood obesity. Clin Chem 2016;62:476–84. 26797688 10.1373/clinchem.2015.249136

[6] Ou Z Wang G Li Q CRTC3 polymorphisms were associated with the plasma level of total cholesterol and the risks of overweight and hypertriglyceridemia in a Chinese Han population. Mol Biol Rep 2014;41:125–30. 24264430 10.1007/s11033-013-2844-4

[7] Wang Y Inoue H Ravnskjaer K Targeted disruption of the CREB coactivator Crtc2 increases insulin sensitivity. Proc Natl Acad Sci U S A 2010;107:3087–92. 20133702 10.1073/pnas.0914897107 PMC2840317

[8] Vega RB Huss JM Kelly DP The coactivator PGC-1 cooperates with peroxisome proliferator-activated receptor alpha in transcriptional control of nuclear genes encoding mitochondrial fatty acid oxidation enzymes. Mol Cell Biol 2000;20:1868–76. 10669761 10.1128/mcb.20.5.1868-1876.2000 PMC85369

[9] Bachman ES Dhillon H Zhang CY betaAR signaling required for diet-induced thermogenesis and obesity resistance. Science 2002;297:843–5. 12161655 10.1126/science.1073160

[10] Wu Z Huang X Feng Y Transducer of regulated CREB-binding proteins (TORCs) induce PGC-1alpha transcription and mitochondrial biogenesis in muscle cells. Proc Natl Acad Sci U S A 2006;103:14379–84. 16980408 10.1073/pnas.0606714103 PMC1569674

[11] Than TA Lou H Ji C Role of cAMP-responsive element-binding protein (CREB)-regulated transcription coactivator 3 (CRTC3) in the initiation of mitochondrial biogenesis and stress response in liver cells. J Bio Chem 2011;286:22047–54. 21536665 10.1074/jbc.M111.240481 PMC3121349

[12] Tsuchida A Yamauchi T Kadowaki T Nuclear receptors as targets for drug development: molecular mechanisms for regulation of obesity and insulin resistance by peroxisome proliferator-activated receptor gamma, CREB-binding protein, and adiponectin. J Pharmacol Sci 2005;97:164–70. 15725703 10.1254/jphs.fmj04008x2

[13] Hardie D Organismal Carbohydrate and Lipid Homeostasis. Cold Spring Harb Perspect Biol 2012;4:a006031. 22550228 10.1101/cshperspect.a006031 PMC3331699

[14] Altarejos JY Montminy M CREB and the CRTC co-activators: sensors for hormonal and metabolic signals. Nat Rev Mol Cell Biol 2011;12:141–51. 21346730 10.1038/nrm3072 PMC4324555

[15] Xing L Gopal VK Quinn PG cAMP response element-binding protein (CREB) interacts with transcription factors IIB and IID. J Biol Chem 1995;270:17488–93. 7615553 10.1074/jbc.270.29.17488

[16] Brickner DG Ahmed S Meldi L Transcription factor binding to a DNA zip code controls interchromosomal clustering at the nuclear periphery. Dev Cell 2012;22:1234–46. 22579222 10.1016/j.devcel.2012.03.012 PMC3376219

[17] Li Y Wang L Zhou L Thyroid stimulating hormone increases hepatic gluconeogenesis via CRTC2. Mol Cell Endocrinol 2017;446:70–80. 28212844 10.1016/j.mce.2017.02.015

[18] Sonntag T Moresco JJ Vaughan JM Analysis of a cAMP regulated coactivator family reveals an alternative phosphorylation motif for AMPK family members. PLoS One 2017;12:e0173013. 28235073 10.1371/journal.pone.0173013 PMC5325614

